# Long-term results of retromuscular hernia repair: a single center experience

**DOI:** 10.11604/pamj.2017.27.132.9367

**Published:** 2017-06-20

**Authors:** Ilker Murat Arer, Hakan Yabanoglu, Huseyin Ozgur Aytac, Ali Ezer, Kenan Caliskan

**Affiliations:** 1 Baskent University Adana Teaching and Research Center, Department of General Surgery, Adana, Turkey

**Keywords:** Fascial closure, incisional hernia, mesh repair, retromuscular repair

## Abstract

**Introduction:**

Incisional hernia (IH) is one of the most frequent postoperative complications after abdominal surgery. There are multiple surgical techniques described for IH repair. The aim of the study is to evaluate the effect of primary fascial closure on long-term results in retromuscular hernia repair (RHR) for incisional hernias.

**Methods:**

A total of 132 patients underwent RHR for IH were included in our study. 109 patients were evaluated in 2009 and 55 patients in 2015 for short and long-term results.

**Results:**

Among 132 patients perfromed RHR, fascia was closed in 107 (81%) and left open in 25 (19%) patients. The mean age of patients was 57.9 ± 11.8 years. Average mesh area was 439.8 ± 194.6 cm^2^, hernia area was 112 ± 77.5 cm^2^ and open area after repair was 40.8 ± 43.3 cm^2^. Mean follow-up of 104 patients regarding postoperative complications evaluated in 2009 was 30.7 ± 14.1 months. Recurrent IH was observed in 6 (4.5%) patients according to data collected in 2009. Long-term results were; mean follow-up period was 91 ± 20.2 months (20-112 months) and recurrent IH was observed in 4 (7.3%) patients.

**Conclusion:**

Retromuscular repair for incisional hernia regardless of the fascial closure gives high patient satisfaction, less recurrence rates and complications in long-term follow-up.

## Introduction

Incisional hernia (IH) is one of the most common postoperative complication after abdominal operations, with an incidence between 11-20% [1, 2]. In high-risk patients such as aortic surgery, this incidence can rise over 35% [3–5]. IH can result in complications such as gastrointestinal obstruction and enterocutaneous fistula [6, 7]. Thus rapid diagnosis and treatment is mandatory for undesirable consequences. There are multiple surgical techniques described in literature. By the invention of prosthetic mesh, there is a trend towards the mesh use for IH. The prosthetic mesh leads improvement in long-term results but the location of the mesh is crucial and it is found to be associated with a high incidence of complications, such as surgical site infection, seroma or gastrointestinal fistula [6, 8, 9]. Positioning of the mesh can be onlay, sublay and inlay. In Chevrel or onlay repair, after dissecting the subcutaneous tissue and approximating 2 edges of the fascia, mesh is placed on the anterior rectus sheath [10]. This can be perfromed when the two edges of the fascia can be approximated. But for larger defects this approximation can be impossible or yield increased tension on the fascia leading to recurrence. There comes in mind another technique described by Ramirez et al. [11], component seperation technique, composed of bilateral release of the external abdominal oblique muscle and fascia, that aids moving the rectus muscles towards the midline to prevent excessive tension. However, component seperation found to be inappropriate for fascial defects >15 cm regarding high recurrence rates [12–14]. There is another alternative technique for component seperation which is described by Rives-Stoppa, sublay repair technique, in which the mesh is placed on the posterior rectus sheath [15]. In a recent meta-analysis, sublay mesh repair is the recommended technique for IH [16]. The retromuscular hernia repair (RHR), the subject of this article, was first described in 1973 [17]. Flament et al. [18] considered this technique as the “gold standard” for midline incisional hernias with a recurrence rate of 6.7 %. Another question for IH repair during RHR was necessity for closure of linea alba. Therefore the aim of this study is to evaluate the effect of primary fascial closure on long-term results in RHR for incisional hernias.

## Methods

**Figure 1:**
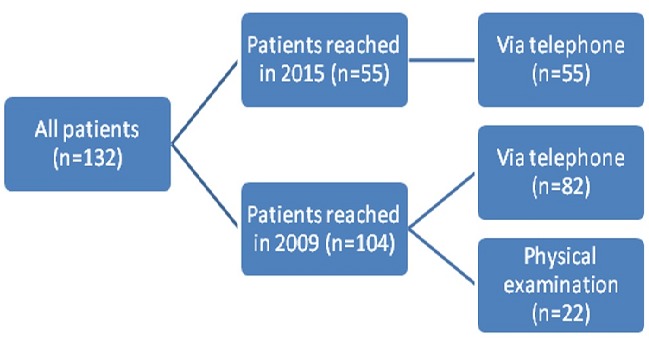
Scheme describing evaluation of patients in 2009 and 2015

**Figure 2:**
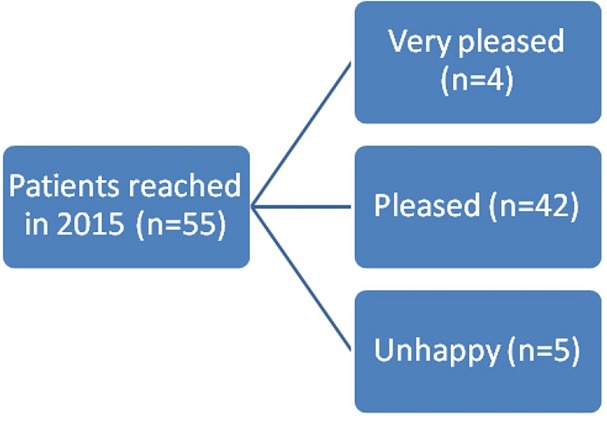
Scheme describing satisfaction level of patients for incisional hernia repair

**Figure 3:**
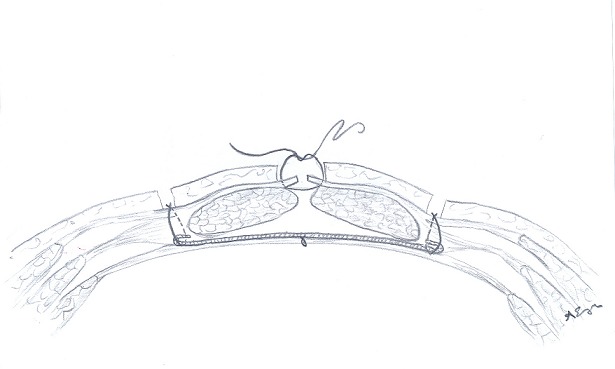
Retromuscular hernia repair with closure of anterior rectus sheath

**Figure 4:**
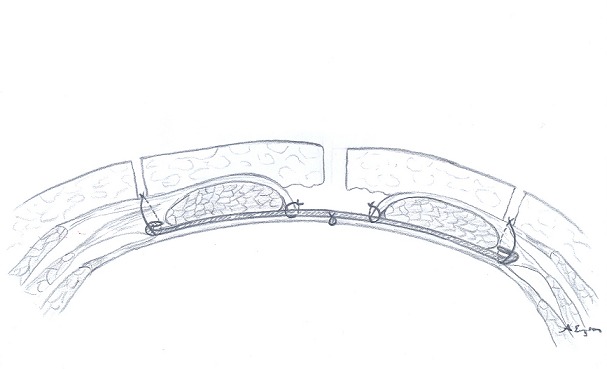
Retromuscular hernia repair without closure of anterior rectus sheath

A total of 132 patients underwent RHR for IH between 2003 and 2009 in Baskent University Adana Teaching and Research center were included in our study. This study is designated as retrospective analysis of a former prospective study to collect the long-term results of IH repair. The data were collected prospectively. Of 132 patients 28 were excluded due to failure to reach the patients. Of 104 patients evaluated in 2009 for early postoperative complications, 82 (78.84%) were evaluated by a questionnaire form applied via telephone and 22 (21.16%) by physical examination (Figure 1). In 2015 a total of 55 patients were reached via telephone, to collect the data of long-term postoperative complications (Figure 2). Verbal informed consent was taken from all patients. In one group the anterior rectus sheath was closed using continuous 1/0 polypropylene sutures (Figure 3), in other group sheath was sutured to the polypropylene mesh where they lay (Figure 4). Statistical analysis was performed with the statistical package SPSS software (Version 17.0, SPSS Inc., Chicago, IL, USA). If continuous variables were normal, they were describle as the mean±standard deviation (p>0.05 in Kolmogorov-Smirnov test or Shapira-Wilk (n<30)), and if the continuous variables were not normal, they were described as the median. Comparisons between groups were applied using Student T test for normally distrubited data and Mann Whitney U test were used for the data not normally distrubited. Values of p < 0.05 were considered statistically.

## Results

Among 132 patients were performed RHR, fascia was closed in 107 (81%) and left open in 25 (19%) patients. 85 (64.3%) were female and 47 (35.7%) were male. The mean age of patients was 57.9 ± 11.8 years (range between 27-82 years). Characteristics of all 132 patients are described on Table 1. The mean body mass index (BMI) was 30.6 ± 5.7 kg/cm^2^. Average mesh area was 439.8 ± 194.6 cm^2^, hernia area was 112 ± 77.5 cm^2^ and open area after repair was 40.8 ± 43.3 cm^2^. 47 (35.6%) patients have co-morbid disease such as; atherosclerosis (13.6%), chronic obstructive pulmonary disease (9.8%), Diabetes Mellitus (8.3%), cancer (3%), hypertension (2.3%) and chronic renal failure (1.5%). 33 (%25) patients had a history of incisional hernia repair. 21 (15.9%) patients had additional operations such as; adhesiolysis (6 patients), cholecystectomy (5 patients), small bowel resection (4 patients), colon resection (2 patients), hiatal hernia repair (2 patients), appendectomy (1 patient), adrenelectomy (1 patient), cholodocoduodenostomy (1 patient) and primary repair of small bowel (1 patient). Mean hospital stay was 4.2 days (min. 1- max. 14 days). Of 132 patients 24 (18.8%) had early postoperative complications. These are; surgical site infection (8 patients), seroma (8 patients), ileus (4 patients), hematoma (3 patients) and suture reaction (1 patient). Mean follow-up of 104 patients regarding postoperative complications evaluated in 2009 was 30.7 ± 14.1 months. Recurrent IH was observed in 6 (4.5%) patients according to data collected in 2009. Among 55 (41.6%) patients evaluated for long-term follow-up, questionnaire applied via telephone in 2015, 17 (30.9%) were male and 38 (69.1%) were female. Mean age was 64.3 ± 11.6 years (range between 36-89 years). Mean follow-up period was 91 ± 20.2 months (20-112 months) and recurrent IH was observed in 4 (7.3%) patients. Verbal questionnaire yield that; 4 (7.3%) patients feel “very pleased”, 42 (76.4%) patients feel “pleased” and 5 (9.1%) patients feel “unhappy”. Effect of fascial closure on complication has been investigated in univariate analysis and found to be not statistically significant (p=0.441). BMI has been investigated as a risk factor for fascial closure and found to have no effect on fascial closure (p=0.421). Hernia area found to be a risk factor for fascial closure (p=0.002) however it has no effect on early postoperative recurrency.

**Table 1:** Characteristics of patients in both groups

	Number of patients	Minimum	Maximum	Mean^+^
Age	132	27	82	57.9±11.8
BMI (kg/cm²)	132	17.5	54	30.6±5.7
Hernia area (cm²)	132	15.7	353.2	112±77.5
Mesh area (cm²)	132	100	900	439.8±194.6
Open area (cm²)	25	5	169.5	40.8±43.3
Hospital stay (Days)	132	1	14	4.2±1.9
Follow-up in 2009 (Months)	104	12	71	30.7±14.1
Follow-up in 2015 (Months)	55	20	112	91±20.2

Abbreviations: BMI; Body mass index.

+ Values are means±standard deviation

## Discussion

Incisional hernias are commonly encountered complications after abdominal operations. They have major complications like intestinal obstruction or enteric fistulas. IH are found to be associated with patient factors such as age, obesity, diabetes and surgical factors such as poor surgical technique and wound infection [19]. Despite advances in surgical techniques IH still have incidence of 11-20% after abdominal operations [1, 2]. The use of prosthetic mesh can be named as a “milestone” in IH repair. If we consider repair techniques before the milestone, the recurrence rates were found to be as high as 31-49 % [20]. Therefore nearly half of the patients with IH repiared primarily have recurrent hernia. Burger et al. [21] found mesh repair superior to suture repair and states that suture repair for IH should be abandoned. The foreign body and fibrosis effect of the mesh lead to decrease in recurrent hernias. As this foreign body has unique advantages, it also possess some disadvantages like wound infection reported between 4-18 % [20]. Since a variety of mesh have been introduced, surgical techniques have also been changed and designated according to placement of the mesh. Onlay, inlay and sublay placement of the mesh have been reported. However onlay repair is believed to easily performed and have less operation time, recently there is a trend towards sublay placement regarding lower recurrence rates [9, 16]. The intra-abdominal pressure may cause fixation of mesh between the posterior fascia and the abdominal muscle and cause reduction in recurrence rates. In a recent review containing 3,945 large incisional hernia repairs with a diameter of 10 cm or a surface of 100 cm2 or more, the use of mesh has better recurrence rates and less hazards and sublay positioning of the mesh is also advised [22]. IH in our study can be classified as large incisional hernias with an average surface of 112 ± 77.5 cm^2^. After the introduction of retromuscular hernia repair by Rives and Stoppa et al. [15] this technique became popular and widely performed by surgeons. By creating a potential space back to rectus muscle, a well-vascularised pocket for mesh can be achieved. Conze et al. [23] found recurrences with RHR technique typically ocur at the upper border and a sublay placement of mesh with an overlap of more than 5 cm to the edges should be performed. Several prosthesis can be used; polypropylene, polyester based and polytetrafluoroethylene mesh. Search for the optimal prosthetic material lead authors to compare light and heavy-weight meshes. However no consensus has been achieved yet.

Introduction of dual mesh that can be placed intraperitoneally, encouraged surgeons to perform laparoscopic repair which yields less scar. Navara et al. [24] compared RHR and laparoscopic incisional hernia repair on 24 patients and found laparoscopic repair safe and feasible but large scaled studies and long-term follow-up should be done. Petro et al. [25] found RHR to be advantageous for the prediction of surgical site occurence. Surgical site infection rates of 0.6 % in the current study is far below literature results unless recurrent IH was observed in 7.3% patients. This paradox suggets us to investigate closure of fascia as a risk factor in development of recurrent disease. Cobb et al. [26] searched for this issue and found fascial closure had no impact on recurrence, surgical site infection and surgical site occurence. We also found leaving fascia open or closed has no impact on recurrence or complications. Long-term follow-up of IH repair (average 97 months for both) similar to average of our study (average 91 months) are evaluated by several studies [27, 28]. However the mesh was placed intraperitoneally in them. In order to evaluate recurrence rates long-term follow-up should be performed. The study of Kruzer et al. [29] has a median follow-up of 84 months near to our study. They also performed sublay repair. Long-term patient satisfaction has been evaluated by few studies. Postoperative discomfort has an incidence of 14-45 % however Kruzer et al. [29] found this ratio to be 6 %. Our findings of patients feeling pleased was 76.4 % that is above Kruzer's findings that was 49 %.

## Conclusion

Incisional hernia repair can be performed by several techniques according to mesh positioning. Retromuscular repair for incisional hernia regardless of the fascial closure gives high patient satisfaction and less recurrence rates and complications in long-term follow-up.

### What is known about this topic

- The retromuscular repair for incisional hernia is the gold standard with a recurrence rate of 6.7 %;
- The effect of fascial closure in retromuscular rapair is still debate;
- Long-term results of retromuscular repair is evaluated by a few studies.

### What this study adds

- Fascial closure has no effect on recurrence or patient satisfaction;
- In long-term follow-up retromuscular repair has a recurrence rate of 7.3 %;
- Patient satisfaction rates are high for retromuscular repair.

## Competing interests

The authors declare no competing interest.
